# 82003 Network Evaluation of a Community-Campus Partnership: Applying a Systems Science Lens to Evaluating Collaboration and Translation

**DOI:** 10.1017/cts.2021.612

**Published:** 2021-03-30

**Authors:** John D. Prochaska, Sharon Croisant, Lesley C. Sommer, Neil Treble, Krista Bohn, Lori Wiseman, Chantele Singleton, Lance Hallberg

**Affiliations:** 1 University of Texas Medical Branch; 2 REACH Coalition of Galveston County

## Abstract

ABSTRACT IMPACT: Using network analysis and a systems science lens, UTMB’s Institute for Translational Sciences is able to quantify the evolution of REACH (its Community-Campus Partnership) as measured by the creation of new partnerships among member entities, promoting the translation and sharing of ideas and resources, and formalization of relationships among members. OBJECTIVES/GOALS: o Present how network analysis and systems science can inform evaluation of community-campus partnerships o Describe results from our experience with evaluating the REACH coalition o Summarize lessons-learned and likely improvements we are considering for our methodology METHODS/STUDY POPULATION: In 2016, we administered a network survey to core members of the Research, Education, and Community Health (REACH) coalition. The survey captured attributes about each organization, including size, populations served, etc. The survey also captured data on the relationships among these organizations, including joint meeting attendance, joint event planning, shared tangible resources, shared information, and formal legal agreements between organizations. These data were analyzed using network analysis methods. The survey was again repeated in 2018, and comparisons were made to evaluate how the network structure had evolved from 2016 to 2018. RESULTS/ANTICIPATED RESULTS: Joint meeting attendance was high in both 2016 and 2018; however, there was evidence of increased sharing of information and tangible resources in 2018. We also observed an increase in joint event planning among partnering agencies. Most strikingly, we observed that the number of formalized agreements (in the form of Memoranda of Understanding or more formalized contracts) between agencies more than doubled between 2016 and 2018. By measuring the evolution of our network of partners, we are able to document the evolution of a community-campus partnership over time. DISCUSSION/SIGNIFICANCE OF FINDINGS: Over the course of 2 years, the coalition signaled an increase in deeper collaborations beyond simply meeting together. The use of network analysis demonstrated utility and provided another dimension for evaluating the development of teams, partnerships, and coalitions.

